# The big chill: Growth of *in situ* structural biology with cryo-electron tomography

**DOI:** 10.1017/qrd.2024.10

**Published:** 2024-12-13

**Authors:** Mikhail Kudryashev

**Affiliations:** 1 In situ Structural Biology, Max Delbrück Center for Molecular Medicine in the Helmholtz Association (MDC), Berlin, Germany; 2 Institute of Medical Physics and Biophysics, Charite–Universitatsmedizin Berlin, corporate member of Freie Universitat Berlin and Humboldt Universitat zu Berlin, Institute for Medical Physics and Biophysics, Berlin, Germany

## Abstract

*In situ* structural biology with cryo-electron tomography (cryo-ET) and subtomogram averaging (StA) is evolving as a major method to understand the structure, function, and interactions of biological molecules in cells in a single experiment. Since its inception, the method has matured with some stellar highlights and with further opportunities to broaden its applications. In this short review, I want to provide a personal perspective on the developments in cryo-ET as I have seen it for the last ~20 years and outline the major steps that led to its success. This perspective highlights cryo-ET with my eyes as a junior researcher and my view on the present and past developments in hardware and software for *in situ* structural biology with cryo-ET.

## Introduction and personal history

In 2005, I was about to complete my physics degree at an institution now called Siberian Federal University (Russia) when EMBL Heidelberg (Germany) invited me to a PhD student interview. My initial intention was to develop myself as a bioinformatician in one of the then-already famous groups; however, Achilleas Frangakis showed me cryo-electron tomography (cryo-ET) and got me excited with the fact that they were noisy, with an anisotropic resolution, and definitely needed computational processing (Frangakis, 2021). However, I failed the final interview to EMBL. Yet, the challenge of cryo-ET and the promise to visualize molecules inside cells at high resolution (Grünewald et al., 2003) were fascinating. Luckily, a malaria biologist, Freddy Frischknecht, recruited me as one of his first PhD students at Heidelberg University (Germany). We collaborated with Wolfgang Baumeister’s department at the Max Planck Institute for Biochemistry, where the pioneering work on establishing cryo-ET was ongoing. This was the start of my cryo-EM journey. My PhD project focused on how *Plasmodium* sporozoites, the infectious stages of the malaria parasite transmitted from a mosquito to a host, move. Unlike most eukaryotic cells, sporozoites are thin enough to be imaged by cryo-ET. The research was exploratory, and we did not know what we were going to find if anything at all. However, as this was potentially a good application of cryo-ET, my co-PI Marek Cyrklaff convinced Wolfgang Baumeister to give me a guest contract to visit MPIB. We froze grids in our lab in Heidelberg, traveled to Munich with a dryshipper, and mostly booked night shifts as this was the only time when the microscope was free. On a good shift, I could record two to four tomograms with a total of ~140 during my PhD. Currently, we can record this much data in one session.

**Figure 1.**
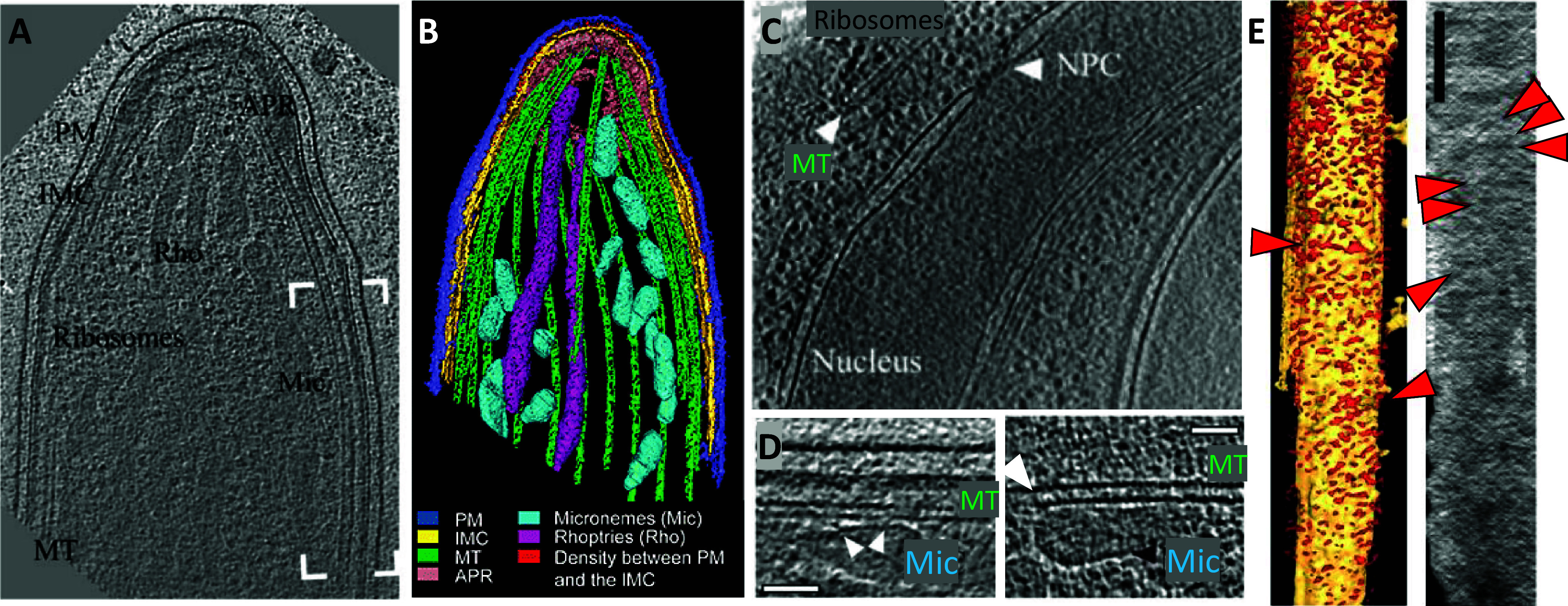
Molecular architecture of the apical end of a Plasmodium berghei sporozoite. (*a, b*) *A slice through a tomogram of the apical end of a sporozoite (a) and its volume rendering (b). The labels point to PM, plasma membrane (blue); IMC, Inner membrane complex (yellow); Rho, Rhoptries (magenta); Mic, Micronemes (cyan); MT, Microtubules (green). Red highlights density between PM and IMC, also shown in e. Adapted from Kudryashev et al. (2010c).* (c) A slice through a tomogram of the central part of a sporozoite showing a part of a nucleus with an apparent nuclear pore complex (NPC), end of a microtubule (MT), and ribosome-looking particles. (d) Slices through tomograms showing a close distance between the microtubules (MT) and micronemes (Mic) with apparent connections. Scale bar: 50 nm. *(e) A volume-rendered visualization of a side view onto the IMC with the removed PM. Red arrowheads point to the filament-like densities where actin filaments are expected. The direction of the electron beam is horizontal. Right: a projection of the EM density through the volume between the IMC and the PM. Scale bar: 100 nm. Panel adapted from* Kudryashev et al. (2010b).

We were successful in the generation of tomographic data and could observe large molecules and organelles inside parasites (Figure 1). By looking at microtubules, we realized that they had an extra density inside (Cyrklaff et al., 2007) suggesting the presence of microtubule inner proteins. These were later identified by Wang et al. in the related parasite *Toxoplasma gondii* (Wang et al., 2021). We discovered that microtubules and most organelles are located at a fixed distance from the parasite’s inner membrane complex, suggesting the presence of a linker molecule (Kudryashev et al., 2010c). Interestingly, parasites, when isolated, glide on solid support preferably in a counterclockwise direction (Hegge et al., 2009). We found that the arrangement of the microtubules around the *Plasmodium*-specific microtubule-organizing center creates a stiff ‘cage’ on the support, on which they glide (Kudryashev et al., 2012). I always found tomograms fascinating – they contain all the information about protein structures in a cell, although at limited resolution. We often observed proteins that we could attribute to textbook molecules, such as ribosomes, nuclear pore complexes, connections between microtubules and transport vesicles (Figure 1c, d), and many unidentified features or features that were at the edge of the resolution limit such as repetitive structures within the polar rings that have now been resolved (Martinez et al., 2022). *Plasmodium* sporozoites move using their own actin machinery and we wanted to visualize it, however, we could never see them under the parasite plasma membrane, where they were expected (Figure 1e). From this, we concluded that they must be rather short (Kudryashev et al., 2010b). Indeed, only recent tomograms revealed their presence (Martinez et al., 2023). Overall, the high-resolution cell biology of *Plasmodium* sporozoites was highly informative and we published the last tomograms 9 years after I defended my PhD (Kehrer et al., 2018) with a number of them remaining unpublished. Recent developments in cryo-EM/ET made the technologies much more accessible, enabling many excellent groups to produce amazing tomograms and structures at higher resolution and gain insights into the molecular architecture of malaria parasites (Anton et al., 2023; Ferreira et al., 2023; Wang et al., 2021; Martinez et al., 2022; Sun et al., 2024) and it is fascinating to see the developments.

In 2005, subtomogram averaging (StA) was implemented as a set of scripts allowing to obtain moderate-resolution structures of molecules in tomograms (Förster et al., 2005), and we had to try it. Yet, we did not find a protein complex that would be interesting, large, and abundant in our tomograms of malaria parasites. We therefore obtained very thin bacteria – spirochetes of the genus *Borrelia*, known for causing Lyme disease. Most bacteria, including spirochetes, move by rotating flagellar filaments by a large transmembrane protein complex called a bacterial flagellar motor (BFM). BFM is a fascinating molecular machine converting proton gradient across the bacterial inner membrane into mechanical rotation. The first structure *in situ* was reported by Grant Jensens’ group (Murphy et al., 2006), it was produced by averaging 20 particles with C16 symmetry and had a resolution of 7 nm. I thought that with more tomograms I could average more particles and improve the resolution. I collected ~35 tomograms over many sessions (Kudryashev et al., 2009) and produced a structure from 128 particles with C16 symmetry at a resolution of 4.6 nm (Kudryashev et al., 2010a). Roughly simultaneously, Jun Liu from Houston produced a much better structure at a resolution of 3.5 nm from a heroic effort of averaging over 1280 particles (Liu et al., 2009). It became clear that obtaining higher resolution structures is possible, and it would require a higher throughput of data collection and processing. Later work with modern equipment and much higher throughput resulted in even higher resolution structures, allowing the fitting of the atomic models of the components of BFM (Carroll et al., 2020; Guo et al., 2022), and there seems to be more potential for further improvement.

When I finished my PhD in 2009, I was lucky to get a postdoc position with Henning Stahlberg at the Biozentrum Basel (Switzerland). Biozentrum had one of the first Titan Krios microscopes, and a part of Henning’s group had a technological inclination. Having access to the automated high-throughput instrument was promising to be able to record large tomographic datasets. At that point, one of the major bottlenecks became data processing – with increased throughput and larger sizes of digital volumes. In 2009, we used Av3 (Förster et al., 2005), a set of MATLAB-based scripts to align subtomograms to one reference on one CPU. It was intuitive, compact, and functional but required a certain computational background to use, and was slow and prone to overfitting, like many other cryo-EM packages at the time (Scheres & Chen, 2012). Another option IMOD/PEET (Nicastro et al., 2006) also worked, it had a graphical interface and was useful for teaching undergraduates, but did not allow the flexibility that we wanted.

Luckily, my office neighbor Daniel Castaño-Diez took the challenge to improve data processing for cryo-ET and StA. He was excited about the potential of GPU acceleration for 3D image processing before it became mainstream (Castaño-Díez et al., 2008). Daniel developed a set of tools now known as Dynamo – a GPU-accelerated package for StA that ran on clusters (Castaño-Díez et al., 2012) and was much faster than CPU-based implementations. Dynamo had a graphical user interface, enabling scientists without coding expertise to use it. Dynamo changed the way we worked: before Dynamo we would start a subtomogram alignment project for an overnight run, and with Dynamo, we could run it over a coffee break. Dynamo widened the bottleneck of StA, but it still required reconstructed tomograms and picked particles to start. Understanding these challenges, Daniel developed variable tools for particle picking and data management in Dynamo Catalogue (Castaño-Díez et al., 2017), which is still very useful in the majority of applications. Daniel first developed Dynamo for our in-house projects, and we naturally wanted others to use it too, and organized many workshops in Basel and around the world. This helped to understand the requirements of Dynamo users; however, the majority of users were onboarded to Dynamo only once it got a wiki page with documentation.

Equipped with the automated microscope and the tools to process data, we were excited to put them to good use. I thought that cryo-ET could make the biggest contribution to the structural analysis of protein complexes that are hard to purify, for example, large membrane protein complexes. We teamed up with Guy Cornelis’s group, prominent researchers in the field of bacterial secretion to determine the structure of the bacterial injectisome in pathogenic *Yersinia enterocolitica.* The injectisome, also known as the type 3 secretion system, is a multiprotein transmembrane complex that bacteria use to deliver their effectors (i.e., toxins) into the host cells. The injectisome is evolutionarily related to BFM (Cornelis, 2006). We generated minicells that were smaller and provided higher contrast and determined a ~40 Å structure of the injectisome in cells. The structure showed significant flexibility of the part of the injectisome located between the bacterial inner and outer membranes, which we attributed to an adaptation to deal with the naturally occurring variations in the intermembrane distance (Kudryashev et al., 2013). The injectisomes tended to form small clusters on the surface of the bacteria, which likely increased the total secretion efficiency (Kudryashev et al., 2015a). We then teamed up with Nans et al. who imaged pathogenic bacteria *Chlamydia* infecting human cells grown on grids. Such imaging naturally contained idle bacteria and those that infected cells, which allowed us to determine the moderate resolution structures of the injectisomes in their inactive and active states. Interestingly, the injectisomes in contact with the host cells had a shorter periplasmic part, suggesting that a ‘pump-action’ is required for secretion (Nans et al., 2015).

At the end of 2013, we got one of the first K2s – a direct electron detector (DED) from Gatan, at that moment – without an energy filter. I was about to join the group of Marek Basler, who just started his group in Biozentrum Basel. The focus of the group was the recently discovered bacterial secretion system type 6 (T6SS), which is similar to an inverted bacteriophage tail (Brunet et al., 2014), which bacteria use to kill each other (Basler et al., 2013, 2012). Marek knew how to purify the T6SS sheath – a helical spring loaded with toxins that stored mechanical energy for a quick release. I prepared cryo-EM grids and collected ~250 micrographs manually over a Saturday on our new system. I have never done single-particle cryo-EM before, especially helical reconstruction, therefore we contacted an expert – Edward Egelman. Within weeks, we got the structure at 3.5 Å resolution (Kudryashev et al., 2015b), which showed most side chains in the ordered part of the protein. In a way, the T6SS sheath is a perfect sample for single-particle cryo-EM as it is very stable and has helical and C6 symmetry. This is why a high-resolution structure could be obtained from the best 77 images. The field was still developing at the time and the software did not have hard blocks on the overfitting of maps, and the building of atomic models was usually done into X-ray maps. Therefore, we had to improvise on the aspects of image processing and building atomic models into the maps *de novo* (Wang et al., 2015). Our early routines were using the data very efficiently, and only a small improvement in resolution from 3.5 to 3.3 Å was demonstrated with the later versions of helical refinement in Relion (He & Scheres, 2017).

A good way to think about structural biology and many other things is the framework of evolution. Max Perutz and John Kendrew solved structures of hemoglobin and myoglobin by X-ray crystallography, and it was a major effort to establish it starting from basic principles (Meurig Thomas, 2020). X-ray crystallography defined structural biology for many years with a method mostly focused on relatively small soluble proteins. To facilitate the crystallization of more difficult targets, a proficient structural biologist had to optimize constructs, truncate domains, and introduce stabilizing mutations, which may limit the functionality of the resulting structures. The entire infrastructure of hardware (from crystallization robots to beamlines) and software for automatic data processing have been developed, making X-ray crystallography a very mature technique. Unwin and Henderson applied the crystallography approach to a crystalline purple membrane containing bacteriorhodopsin using electrons as probes (Henderson & Unwin, 1975). This gave rise to a method of electron crystallography, motivating many researchers to grow 2D crystals of membrane proteins and image them in diffraction or imaging modes on an electron microscope (Abeyrathne et al., 2012). This technology was moderately successful, resulting in structures of many membrane proteins including 1.9 A structures of AQP0 (Gonen et al., 2005) and interestingly, tubulin in a lattice (Nogales et al., 1998). However, as 2D crystals of membrane proteins are generally hard to grow and screen, the method has become less popular over time. Small 3D crystals can be analyzed by microcrystal electron diffraction (microED), and the larger crystals can even be FIB-milled to reduce their thickness (Mu et al., 2021), however, it still requires crystallization. Nuclear magnetic resonance (NMR) has also evolved as a structural biology method that is very successful for the structural analysis of proteins, including membranes. NMR requires a high concentration of stable protein and is limited in the sequence length (Marion, 2013). Single-particle cryo-EM solved most of the problems of the existing methods: it required much less protein, and the protein did not have to be 100% pure and stable for days. Single-particle cryo-EM does not require crystallization, reveals structures of proteins in solution, and allows us to understand the structure and dynamics of macromolecules from one dataset. Eventually, electron microscopists transitioned from niche structural biologists studying ‘blobs’ to the ‘superstars’ of science, culminating with the award of the Nobel Prize in Chemistry in 2017 to the pioneers of cryo-EM. Single-particle cryo-EM was particularly successful in determining structures of membrane proteins and ion channels with the quintessential structure of the TRPV1 channel (Liao et al., 2013), as previously they were very hard to crystallize.

Inspired by single-particle cryo-EM, in 2015, I made a detour from cryo-ET and started a research group focusing on the structure and function of ion channels, which I thought were understudied and seemed like a good target for cryo-EM. With the cryo-EM hype and some luck, I got a big grant from the Alexander von Humboldt Foundation and recruited an amazing team to the Max Planck Institute of Biophysics in Frankfurt on Main. Notsurprisingly, membrane protein biochemistry proved to be hard: mammalian protein expression and purification are often very challenging and require a lot of optimization. Even when it works, there are day-to-day variability. A major challenge in determining the structure of a membrane protein is, therefore, not the cryo-EM work, but the quality of the protein. As a result, the competition in the field of cryo-EM of membrane proteins is immense, as many outstanding labs generate bright ideas and stable pure proteins. As a postdoc, I analyzed the structure of the serotonin receptor ion channel 5-HT3R, which instead of growing into 2D crystals, preferred to end up in small lipid vesicles. I, therefore, used cryo-ET and STA and determined the structure of 5-HT3R in lipids at a moderate resolution of 12 A. The structure was very similar to the previously reported X-ray structure at this resolution (Kudryashev et al., 2016). However, high-resolution single-particle analysis of 5-HT3R in a membrane mimetic saposin determined by Yingyi Zhang and Patricia Dijkman showed major differences from the previous structures without lipids. Functionally important cholesterol stabilizes the structure of 5-HT3R and together with phospholipids allows the ion-selective pore in the membrane to open and conduct ions (Zhang et al., 2021). Our groups determined several other important structures: together with the late Herman Bujard, we determined the structure of the malaria surface protein MSP1 (Dijkman et al., 2021), which he developed over 40 years into a malaria vaccine candidate currently undergoing clinical trials (Blank et al., 2020). Together with Volker Haucke, we determined the structure of a membrane-associated kinase PI3KC2α, which allowed us to suggest the mechanism of its action (Lo et al., 2022).

As amazing as single-particle cryo-EM is, I find that membrane protein biochemistry is a critical bottleneck. Depending on the purification protocol, the use of different detergents or membrane mimetic, lipid composition, and others, the structures can be different (Dalal et al., 2024; Hoffmann et al., 2024). Therefore, the structures need to be critically evaluated for their physiological relevance. In my opinion, at the next iteration of structural biology, we can determine the structures of membrane proteins in membranes by skipping protein purification steps. This will allow us to obtain unambiguous structures of membrane proteins in lipid bilayers, potentially with the interacting partners or under physiological gradients. Our group at the Max Delbruck Center for Molecular Medicine in the Helmholtz Society in Berlin pushes ‘ways to means’ to determine the structures of membrane proteins in unrestricted membranes. For this, we experiment with display systems, from cryo-FIB milling into cells followed by cryo-ET, or by analyzing membrane proteins in purified native membranes, such as the vesicles of sarcoplasmic reticulum purified from rabbit muscle (Chen & Kudryashev, 2020; Sanchez et al., 2020) or synaptic vesicles purified from mouse brains (Kravcenko et al., 2024). As the structure determination methods are still being developed, it is clear that high-throughput and streamlined processing will be needed to average over many thousands of asymmetric particles; therefore, we develop automation tools, such as tomoBEAR (Balyschew et al., 2023). In some cases, the structures of proteins in membranes can be determined by single-particle cryo-EM, without recording tomograms (Mandala & MacKinnon, 2022; Tao et al., 2023; Yao et al., 2020); however, there seem to be limits to the protein size and flexibility. Perhaps, hybrid StA (Sanchez et al., 2020; Song et al., 2020), combining some advantages of both imaging modalities will be useful for a class of proteins? We work on it while aiming to produce new insights into transmembrane signaling by medically important membrane proteins.

## How cryo-ET and StA became great: Hardware and the interplay between academia and industrial developments

Conceptually, cryo-ET is simple: a sample, protein solution, or small organisms are frozen in a thin layer of amorphous ice; consecutive tilted transmission images are recorded, computationally aligned, and reconstructed into a 3D volume; multiple copies of the same molecules can be identified, mutually aligned and averaged (Walz et al., 1997). However, the actual implementation was challenging. Tomograms have to be collected keeping the total electron dose limited, after each tilt of the microscope stage, the position of the sample has to be re-centered, and refocusing needs to be performed. Although heroes of early cryo-ET could collect tilt series manually (Cheng et al., 2007), making cryo-ET practically useful required automation. The first tomography microscope at the Max Planck Institute for Biochemistry was a Phillips CM300 with a motorized ‘compuStage’, a CCD camera Gatan US1000, and a side-entry holder operated by in-house developed software. Side-entry holders are generally not designed to be tilted to 60°; therefore, liquid nitrogen used to cool it down started bubbling, pouring out at high tilts, and required refilling with liquid nitrogen every few hours. Replacing the grids with such a system took a significant time and was associated with diminishing the microscopes’ vacuum, practically limiting the throughput to three grids per day. Following this successful prototype, FEI (later purchased by Thermo Fisher Scientific) and JEOL designed more stable cold stages with multigrid capabilities. FEI Polara had a multispecimen holder, which was still hard to use, and it was easy to drop a cartridge with a grid inside the microscopy column. More modern microscopes with an autoloader are automated and allow to screen up to 12 grids per loading, which could be done more than once per session, it is a big factor for throughput.

The microscopes and cameras are fully computer-controlled, and the microscope producers maintain application programming interfaces (APIs) to allow external developers to control the devices. This open environment enabled academics to develop creative ideas for data collection, such as the automation packages Apion for single-particle cryo-EM (Lander et al., 2009), TOM toolbox (Nickell et al., 2005), Leginon for tomography (Suloway et al., 2009), and the commonly used SerialEM (Mastronarde, 2005; Schorb et al., 2019). Building on the scripting capabilities of SerialEM enabled further improvements in tomography such as the dose-symmetric tilt scheme (Hagen et al., 2017), which enabled the Briggs group to obtain the first sub-4 Å structure by cryo-ET (Schur et al., 2016). Scripting further enabled recording tomograms in parallel (Bouvette et al., 2021; Eisenstein et al., 2023; Khavnekar et al., 2023), greatly increasing the throughput of data collection. This is borderline revolutionary and switched the challenge of cryo-ET from having enough time on the microscope to record the data to have enough computational resources to store and process all the data. Therefore, it is highly beneficial for both academic researchers and the industry to maintain APIs to further collaborate on innovations.

The detector quantum efficiency (DQE) of photographic film was generally comparable to or better than that of the CCD cameras of the early 2000s (McMullan et al., 2009; Ruskin et al., 2013). There were first-generation FEI Titan Krios with chambers for photographic film, suggesting that some of the instruments were meant to screen the grids using CCD and to collect the data on film. The current generations of DED much surpass the DQE of film and provide high data collection speed, allowing recording ‘movies’ and correcting beam-induced motion (BIM; Brilot et al., 2012; Zheng et al., 2017). The development of DEDs is considered the major factor in the breakthrough of single-particle cryo-EM (Nogales, 2016). However, I would argue that for cryo-ET, without the parallel developments of automated microscopes, computer hardware, and software, the progress would not have been as impressive. As I discussed earlier, the automation of microscopy is much more important for tomography and it was already implemented on CCD cameras, DED gave a large boost in the resolution of final structures. I think that it is important to give credit to the industrial research and development teams who invested time and money to produce hardware at scale and robustly. My only concern with hardware and commercial software is that we need to ensure significant competition in the markets of microscopes, cameras, and software to keep the prices competitive for the limited research budgets.

The current frontiers of hardware technology include sample preparation for cryo-ET, especially for interesting and challenging samples ranging from cells to tissues. Following the pioneering developments of Marko et al. (2007), the department of Wolfgang Baumeister turned the milling of cryo-preserved samples with ions into a robust technology. Currently, milling into individual cells has become a commercially available technology. Further developments by the groups of Juergen Plitzko, Julia Mahamid, Alex Noble, Michael Grange, and others target the methods to image complex samples and multicellular organisms (Schiøtz et al., 2023; Matsui et al., 2024). While currently, it seems difficult, some of these technologies will become routinely useable sooner rather than later. Another potentially large step for cryo-ET is the development of a laser phase plate by the group of Holger Muller, which will allow the recording of data close to focus (Schwartz et al., 2019), further improving the quality of tomograms.

## How cryo-ET and StA became great: Rise of software and software workflows

**Figure 2.**
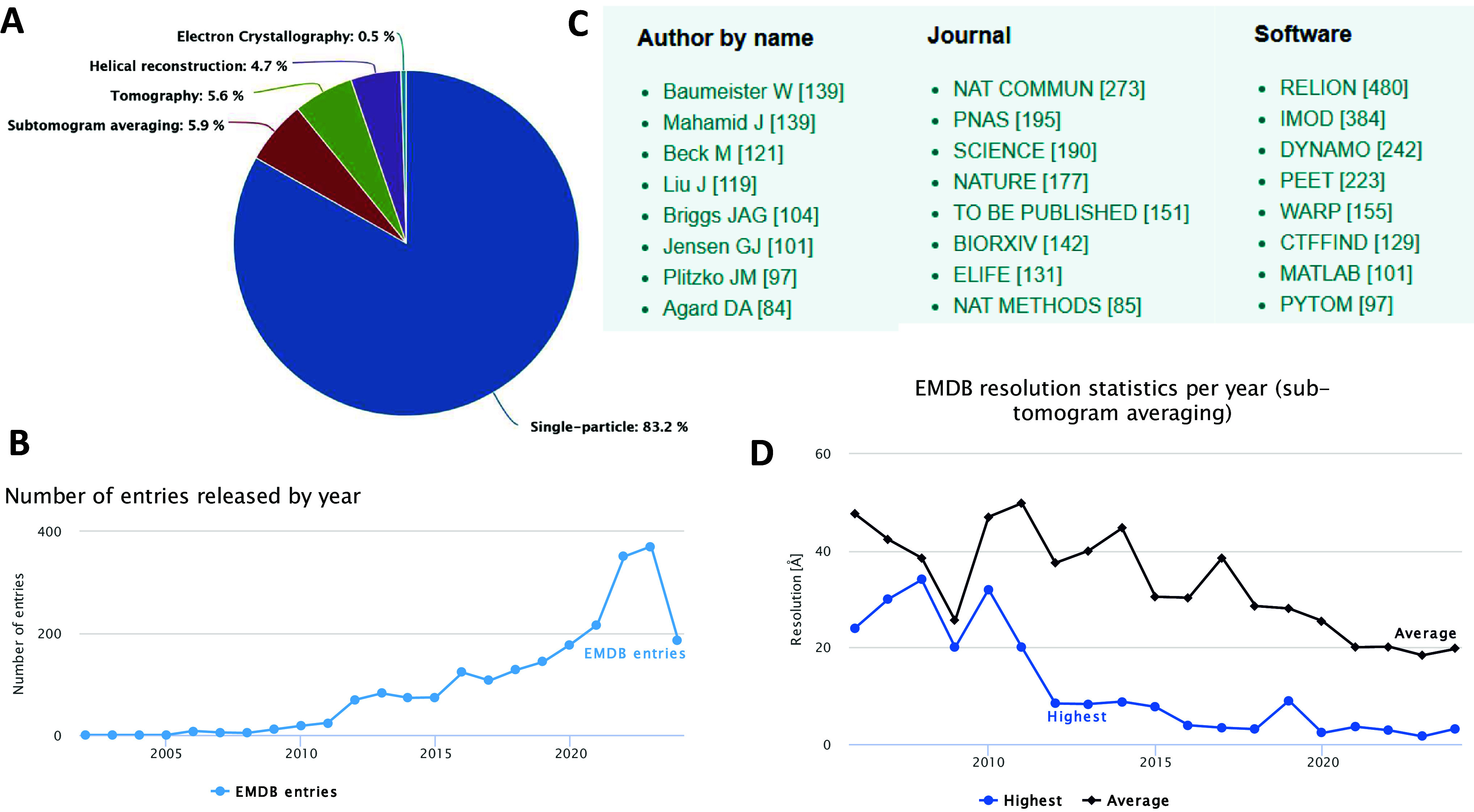
Statistics of EMDB depositions for subtomogram averaging in July 2024.

In July 2024, the EMDB depository (the wwPDB Consortium, 2024) had ~2185 subtomogram averages, slightly under 6% of all the depositions. The number of depositions grows fast and the resolution of the structures improves with years (Figure 2). The top 200 depositions have the resolution of 6.7 Å or higher. Such resolution allows for the resolving alpha-helices and a reasonable positioning of available atomic models. Many papers report subtomogram classifications resulting in multiple depositions of the same molecule from the same datasets. It highlights the power of cryo-ET and StA in the hands of several groups. There are many ‘favorite molecules’ of subtomogram average reports: purified apoferritin, lattices of rotaviruses such as GAG (Obr et al., 2022; Schur et al., 2016), and ribosomes (Gemmer et al., 2023; Tegunov et al., 2021; Xing et al., 2023). I would also note actin filaments from sarcomeres *in situ* (Wang et al., 2022), microtubules in axonemes (Tai et al., 2023) an archeal S-layer (von Kügelgen et al., 2024), and a COPII coat (Hutchings et al., 2021). Highest resolution structures typically contain a large number of particles: 35,061 particles for a purified ribosome at 3.1 Å (EMD 33834); 286,400 particles for a ribosome in cells (3.1 Å resolution, EMD-16721); and 77,659*C6 for the CA-SP hexameric lattice at 2.9 Å resolution (EMD-14013). These are pioneering high-resolution structures, and except for apoferritin, provide insights into the structure of molecular complexes *in situ.* Interestingly, many deposited structures were part of publications in reputable journals (Figure 2).

I attribute the broader adoption of structural analysis by StA at higher resolution to the developments in software, which I break down into two 2 factors. The first factor – the software has become more usable. Many academic packages are good at doing one or a few operations. However, interfacing software responsible for multiple steps is not trivial and often requires scripting input and output to sometimes poorly documented packages. We previously estimated that processing of cryo-ET and StA datasets may include up to 16 steps of various difficulty and computational complexity (Leigh et al., 2019). To my knowledge, many successful StA labs had in-house solutions based on MATLAB or bash scripts, which are hard to maintain or modify for the new versions of infrastructure or other packages. Such sets of scripts are hard to transfer to another system, and there is a risk that once a scientist who wrote them leaves the group – they will be unusable. The development of workflows, covering multiple steps of data processing, such as IMOD/PEET (Heumann et al., 2011; Mastronarde & Held, 2017), EMAN2 (Chen et al., 2019), pyTom (Hrabe et al., 2012), emClarity (Ni et al., 2022), Dynamo (Scaramuzza & Castaño-Díez, 2021), Warp-Relion-M (Tegunov et al., 2021), TomoBEAR (Balyschew et al., 2023), nextPYP (Liu et al., 2023), Relion5 (Burt et al., 2024), TOMOMAN (Khavnekar & Wan, 2024), and others makes the processing more accessible. There are many advantages of using a workflow, among others: potentially optimized execution with the reduced requirement for computational resources and storage; more reliability and reproducibility, better documentation, and an easier learning curve for new researchers. To obtain thousands to hundreds of thousands of asymmetric units for averaging to obtain a high-resolution structure, a large degree of automation is required.

The second aspect of the software is the opportunity to refine the structures to high resolution, making the method attractive to many biological applications. Here, two main challenges exist: computational costs and the accounting for the beam-BIM. Large computational resources are spent for the maximization of 3D cross-correlation (CC): a template is rotated to a set of angles, filtered, and distorted by a missing wedge, and the CC between it and the particle is calculated in 3D by convolution in Fourier space. The maximal CC value determines the optimal angles, whereas the position of the CC peak determines the shifts. Importantly, multiple 3D rotations and 3D Fourier transforms need to be calculated to perform CC maximization. When reaching high resolution in StA, the number of informative voxels in a volume increases, increasing the size of the ‘box’ with the particles. Practically, the largest currently deposited structures in EMDB are in the range of 400 voxels^3^, further processing is limited by the memory of graphical processors and, given the need to average over large datasets, the speed of processing altogether. This limitation can be partially circumvented by reducing the calculator of 3D CC to a set of 2D CCs. In the context of cryo-ET, it was first suggested by Ricardo Sanchez from our group (Sánchez et al., 2019a, 2019b) and used in high-end processing packages such as ‘M’ (Tegunov et al., 2021) and Relion 4/5 (Burt et al., 2024), reducing the computational complexity of the algorithm from L^3^ to L^2^, where L is the size of the volume. Still, further improvements in algorithms and their implementations are needed to reduce the computational and environmental load of StA and to make StA accessible to more groups that do not have access to large computational resources.

Accounting for BIM in tomography is arguably more difficult than in 2D single-particle cryo-EM, as the motion is 3D (Brilot et al., 2012; Zheng et al., 2017) and tomographic imaging is performed at tilts. Furthermore, cryo-ET imaging is performed on something more difficult than purified proteins, therefore the motion of the sample could be non-trivial, although it is not well described. Tomography-specific implementation of motion correction by ‘constrained single-particle-cryo-ET’ was first suggested by Bartesaghi et al. (2012). Alignment parameters of the tilt series were refined based on their fit to the final structure, resulting in a ~ 8 Å structure of GroEL from CCD data. This elegant algorithm has not been utilized for a decade, and it was recently reimplemented in a workflow nextPYP (Liu et al., 2023). A similar approach was suggested in *emClarity*: local refinement of tilt series alignment parameters based on the fit of the particles in 2D projections to the final structure, leading by iterative refinement (Himes & Zhang, 2018). ‘M’ by Tegunov et al. further improved the BIM refinement by incorporating several distortions for several types of molecules into one cost function and introduced an elegant filtering of half-maps during subtomogram alignment (Tegunov et al., 2021). EMAN2 introduced the direct ‘subtilt’ refinement of 2D projections and CTF parameters of particles versus the structure (Chen et al., 2019). Relion 4/5 introduced Bayesian priors for motion between the neighboring particles in tomograms to prevent overfitting, resulting in improved reliability & precision (Burt et al., 2024).

As powerful as cryo-ET is for the highlight applications, in my opinion, the range of molecules potentially amenable for the analysis by StA is still limited to the ‘favorite molecules’ which are stable, abundant, and large or periodic assemblies. Such molecules can be identified by automatic algorithms (Chen et al., 2017; de Teresa-Trueba et al., 2023; Moebel et al., 2021), can be easily computationally aligned to templates, and further refined to high resolution. In a way, there are similar limits of molecular weight to be able to identify a particle of interest and to align it to a template. However, an average protein in a cell is ~50 kDa, as a monomer (Kozlowski, 2017), which is much smaller than the highlighted applications and is present in cells (and tomograms) in low copy numbers (Wiśniewski et al., 2014). Alignment of particles to an average is a particular challenge for membrane proteins and the membrane has high contrast (Chang et al., 2023). In fact, the average structure deposited to EMDB in 2024 has a resolution of ~20 Å. Such structures are also highly useful, especially as atomic models of proteins and complexes can be predicted by Alphafol3 (Abramson et al., 2024) or other approaches.

## Future of *in situ* structural biology with cryo-ET: a broadly used method for biological discovery

Methods such as X-ray crystallography, and single-particle cryo-EM matured somehow similar to cryo-ET. At first, the basic concepts and prototype hardware and software were developed by pioneers, followed by the commercialization or open-sourcing of the tools, making the technologies generally accessible. I believe that currently *in situ* structural biology with cryo-ET passed the early steps of reaching general accessibility. Sample preparation and microscopes have become robust and highly productive. Importantly, several initiatives enabled shared access to microscopes (Zimanyi et al., 2022), such as EMBL, NECEN, eBIC, Simons Electron Microscopy Center, and NIH. This means that any researcher can record several hundreds of high-quality tomograms of their sample of interest for a reasonable fee under the supervision of an expert. In the future, hardware will improve with newer detectors and phase plates; such add-ons would be easier to purchase and manage by larger cryo-EM/ET centers.

The major bottleneck for a new group interested in joining the cryo-ET community is data processing. Starting from storing terabyte-scale datasets, current software generally has a steep learning curve and requires large computing resources. The developments in making the software more stable, computationally efficient, and generally usable would provide benefits to the adoption of cryo-ET as a mainstream method. Graphical user interfaces and extensive documentation, like in IMOD, EMAN2, Dynamo, Relion5, and nextPYP, are key to enabling the users to take full advantage of the cryo-ET data. Importantly, many of the tasks in cryo-ET and StA are performed by conventional deterministic algorithms, such as CC-based tilt series alignment, template matching, subtomogram alignment-based CC maximization, and subtomogram classification using flavors of multireference alignment. Many of these operations can be performed by neural networks that can be either pretrained on large datasets or/and trained on a small part of the dataset of interest. Neural network inference is can be faster than CC, which will make processing less computationally demanding. Such implementations of neural networks would work best as a part of an integrated workflow.

Ultimately, as hardware and software will become better, we will be able to see more details in tomograms. On thin samples, perhaps from milled preparations, we will be able to observe molecules and individual domains *in situ.* Annotating 3D volumes manually is difficult and we will need to use algorithms to build atomic models of macromolecular complexes into tomograms. For this purpose, complementary information about protein structure and flexibility, protein–protein interactions, cellular localization, and other factors could be integrated into dedicated multimodal neural networks. Identified proteins could be further processed by StA. Such molecular mapping *in situ* will enable an understanding of the molecular landscapes of cells in high-resolution detail, which will become an important tool in molecular systems biology. In the future, understanding the cellular landscapes in detail will provide numerous insights into the molecular mechanisms of life and could allow for analyzing disease scenarios and suggesting therapeutic strategies.
