# Supplementary material for: The big chill: Growth of in situ structural biology with cryo-electron tomography
Source: QRB Discov. 2024 Dec 13;5:e10. doi: 10.1017/qrd.2024.10 (PMC11649376; doi:10.1017/qrd.2024.10)
Supplement: Peer review history

# Editor report: Decision: The big chill: Growth of in situ structural biology with cryo-electron tomography — R0/PR2

**DOI:** 10.1017/qrd.2024.10.pr2

**Affiliations:** Chemistry, Chalmers University of Technology, Sweden

No accompanying comment.

# Editor report: Decision: The big chill: Growth of in situ structural biology with cryo-electron tomography — R1/PR4

**DOI:** 10.1017/qrd.2024.10.pr4

**Affiliations:** Chemistry, Chalmers University of Technology, Sweden

No accompanying comment.
